# A Randomized Controlled Trial on Analgesic Effects of Intravenous Acetaminophen versus Dexamethasone after Pediatric Tonsillectomy

**DOI:** 10.5812/ircmj.9267

**Published:** 2013-11-05

**Authors:** Seyed Hamid Reza Faiz, Poupak Rahimzadeh, Mahmoud Reza Alebouyeh, Minow Sedaghat

**Affiliations:** 1 Department of Anesthesiology and Pain Medicine, Rasoul-Akram Medical Center, Tehran University of Medical Sciences (TUMS), Tehran, IR Iran

**Keywords:** Tonsillectomy, Acetaminophen, Dexamethasone, Analgesia

## Abstract

**Background:**

A few studies are available actually comparing the clinical efficacy of intravenous acetaminophen with other medications such as dexamethasone to inhibit postoperative adverse events in children.

**Objectives:**

This randomized blinded controlled trial was designed to compare controlling status of postoperative events in children after tonsillectomy randomized to receive either intravenous acetaminophen or dexamethasone.

**Patients and Methods:**

Eighty four children aged between 4 to 13 undergoing tonsillectomy were randomized using a computer-generated schedule to double-blind treatment with intravenous acetaminophen (15 mg/kg) or intravenous dexamethasone (0.1 mg/kg). Children were post-operatively assessed for swallowing pain, pain while opening mouth, ear pain, and postoperative sore throat in recovery room (within one hour after surgery), at the time of admission to the ward, as well as at 12 and 24 hours after surgery, assessed by the objective pain scoring system (OPS; minimum score: 0 = no pain, maximum score: 10 = extreme pain).

**Results:**

There were no significant differences between the two groups with regard to the severity of postoperative pain due to swallowing or opening mouth measured at the different study time points from postoperative recovery to 24 hours after the surgery. There was no difference in ear pain severity at the time of postoperative recovery, at the admission time to ward and also at 12 hours after surgery; however mean score of ear pain severity was significantly higher in those who administered acetaminophen 24 hours after operation. Also, the mean score severity of sore throat was significantly higher in the acetaminophen compared with the dexamethasone group within 12 hours of surgery. Postoperative vomiting and bleeding were similarly observed between the two study groups. The severity of swallowing pain, pain while opening mouth, ear pain, as well as postoperative sore throat as gradually assuaged within 24 hours of tonsillectomy in both groups, however no between-group differences were observed in the trend of the severity of these events.

**Conclusions:**

The dexamethasone-based regimen may have more advantage over the intravenous acetaminophen regimen for inhibiting pain and PONV following tonsillectomy in children.

## 1. Background

Tonsillectomy is one of the most frequent surgical procedures carried out in children which is commonly associated with increased risk of some complications such as severe pain while swallowing and postoperative nausea and vomiting (PONV) (1). Poorly controlled pain and PONV are the most common reasons delaying discharge home. Thus, these events should be prevented and treated using a multimodal approach including selection of a suitable anesthetic technique. The postoperative events can be usually prevented and removed by administrating a combination of small dosages of opioid analgesics and anti-inflammatory drugs (2-4). In turn these drugs might lead to increased risk of some adverse phenomena such as postoperative bleeding and respiratory depression (5, 6). In this context, the use of intravenous acetaminophen is currently considered as the basic postoperative analgesic due to its low cost and an appropriate safety profile in the management of post-tonsillectomy pain (7). Furthermore, parenteral formulation of acetaminophen has been introduced for children and its pharmacological safety has been established among this age-subgroups (8, 9).

Dexamethasone is the other drug used for diminishing postoperative pain and PONV in various operations. The effects of the dexamethasone on decreased edema and fibrosis have been clearly substantiated. The anti-inflammatory mechanism of corticosteroids is considered to be a result of the inhibition of phospholipase A2, the crucial membrane enzyme regulating the cascade for the production of hyperalgesic leukotrienes and prostaglandins from arachidonic acid in response to inflammatory stimuli (10-14), the use of dexamethasone in both children and adults have produced conflicting results with respect to the effects on pain and morbidity following tonsillectomy.

There are few studies truely comparing the clinical efficacy of intravenous acetaminophen with other medications such as dexamethasone. This randomized blinded controlled trial was designed to compare controlling status of postoperative events in children after tonsillectomy randomized to receive either intravenous acetaminophen or dexamethasone.

## 2. Objectives

This randomized blinded controlled trial was designed to compare controlling status of postoperative events in children after tonsillectomy randomized to receive either intravenous acetaminophen or dexamethasone.

## 3. Materials and Methods

### 3.1. Study Population

This study was a randomized controlled trial with blinded assessment of postoperative pain severity and nausea and vomiting (PONV). Children were eligible if they were undergoing tonsillectomy, aged between 4 and 13 years and of ASA physical status I to II. Exclusion criteria were any renal or hepatic impairment, neurological disorder impairing accurate pain assessment, sensitivity to any trial drug, requirement for premedication and other analgesics administered on the day of surgery. After institutional Ethics Committee approval and informed consent from a parent in each case, 84 patients were randomized using a computer-generated schedule to double-blind treatment with intravenous acetaminophen 15 mg/kg (Acetaminophen 1gr/6.7mL - UNIPHARMA S.A manufactured in E.U) or intravenous dexamethasone 0.1 mg/kg (8mg/2mL – DAROU PAKHSH IRAN). Drugs were administered in 50 ml normal saline while using micro set and infused over 30 minutes. Study drugs were marked only with coded number labels and administered within the last 30 minutes of the surgical procedure.

### 3.2. Anesthesia Technique

At operation room and after setting ECG monitoring, non-invasive blood pressure, capnography, and pulse oxymetry, intravenous line was secured with 22G i.v canula and ringer lactate infusion (8 to 10 cc/kg) was started. All patients were uniformly premedicated with Midazolam 0.05 mg/kg, and Fentanyl 2 µg/kg IV. After preoxygenation with 100% O2, anesthesia was induced with sodium thiopental (5 mg/kg) and Cisatracurium (0.15 mg/kg) and maintained with Sevofluran 2.5%. After the three minutes, patients were intubated endotracheal tube without cuff but tailored to the patients’ age and weight. All intubations were performed by an experienced anesthesiologist. After surgery, all patients were transferred to the recovery room. The mean duration of surgery was 45 minutes.

Study Parameters Measurement: Children were postoperatively assessed for swallowing pain, pain when opening mouth, ear pain, and postoperative sore throat in recovery room (within one hour after surgery), on the time of admission to the ward, as well as at 12 and 24 hours after surgery, assessed by the Objective pain scoring system (OPS; minimum score: 0 = no pain, maximum score: 10 = extreme pain). The incidence of PONV and postoperative bleeding was also assessed postoperatively. Our study outcome measure was the incidence of postoperative pain severity as well as PONV and bleeding. After surgery and in the ward, children with OPS ≥ 5 medicated with meperidine PRN (0.3 mg/kg). In the cases with the appearance of vomiting, ondansetron PRN (0.1 mg/kg) was administered. Also, after establishment of oral feeding, Ibuprofen syrup (6 mg/kg/q8h) was administered.

### 3.3. Statistical Analysis

A sample size calculation was performed using a change in OPS mean values of 2.0 with a SD of 1.5. Calculations were performed to determine the number of patients required in this study to have 80% power to detect as statistically significant (P < 0.05). By accepting a type I error of 0.05 and a type II error of 0.20, the study size needed was 40 patients per group. Results were reported as mean ± standard deviation (SD) for the quantitative variables and percentages for the categorical variables. The groups were compared using the Student’s t-test or the Mann-Whitney U test for the continuous variables and the chi-square test (or Fisher’s exact test if required) for the categorical variables. Trend of the changes in continuous variables was compared between the groups using the repeated measure for ANOVA test. This study was done with the power of 80%. P values of 0.05 or less were considered statistically significant. All the statistical analyses were performed using SPSS version 16.0 (SPSS Inc., Chicago, IL, USA).

## 4. Results

From a total of 84 eligible patients that were invited to participate, all of them completed the trial (42 patients in the Acetaminophen group and 42 cases in the dexamethasone group). Average age of all participants was 7.07 ± 2.22 years, median 7.00 years, ranged 4-13 years). The two interventional groups received acetaminophen or dexamethasone were matched in terms of mean age (6.81 ± 2.28 years versus 7.33 ± 2.15 years, P = 0.281) and frequency of male gender (59.5% versus 64.3%, P = 0.653). As summarized in Table 1, there were no significant differences between the two groups with regard to the severity of postoperative pain due to swallowing or opening mouth measured at the different study time points from postoperative recovery to 24 hours after the surgery. Regarding appearance and severity of ear pain, there was no difference in pain severity on the time of postoperative recovery, on admission time to ward and also at 12 hours after surgery; however mean of pain severity score was significantly higher in those who administered acetaminophen compare to another group at 24 hours after operation. Also, with respect to postoperative sore throat, the mean severity of this complication was significantly higher in the acetaminophen compared with the dexamethasone group within 12 hours of surgery.

**Table 1.** Differences Between the two Groups With Regard to the Severity of Postoperative Pain

Item	Acetaminophen group (n = 42)	Dexamethasone group (n = 42)	P-value
**Swallowing pain (mean/SD)**			
On post-operative recovery	4.36/1.92	4.19/1.53	0.662
On admission to ward	3.45/1.67	3.14/1.26	0.341
12 hours after surgery	2.31/1.72	2.26/1.40	0.890
24 hours after surgery	1.93/1.77	1.71/1.57	0.560
**Pain when opening mouth (mean/SD)**			
On post-operative recovery	3.36/2.25	3.50/1.66	0.741
On admission to ward	2.43/1.94	2.33/1.54	0.804
12 hours after surgery	1.71/1.73	1.64/1.45	0.838
24 hours after surgery	1.26/1.62	1.24/1.61	0.946
**Ear pain (mean/SD)**			
On post-operative recovery	1.60/1.94	1.31/1.70	0.475
On admission to ward	1.17/1.50	0.81/1.19	0.230
12 hours after surgery	0.76/1.19	0.43/0.77	0.131
24 hours after surgery	0.48/0.97	0.07/0.26	0.012
**Postoperative sore throat (mean/SD)**			
On post-operative recovery	4.95/1.61	4.02/1.72	0.012
On admission to ward	3.67/1.59	2.90/1.57	0.030
12 hours after surgery	2.88/1.76	2.17/1.40	0.042
24 hours after surgery	2.17/1.78	1.98/1.47	0.595

**Figure 1.**
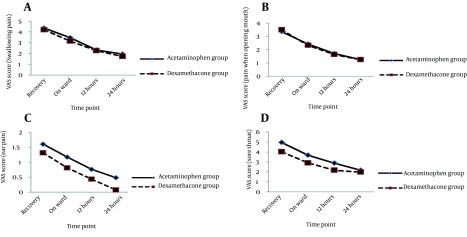
Trend of the changes in postoperative events in the groups received intravenous acetaminophen or dexamethasone A: comparison of the Swallowing pain between the acetaminophen groups versus the Dexamethasone groups during 24 hours after the end of the surgery. B: comparison of the pain when the patient opens his or her mouth between the acetaminophen groups versus the Dexamethasone groups during 24 hours after the end of the surgery. C: comparison of the ear pain between the acetaminophen groups versus the Dexamethasone groups during 24 hours after the end of the surgery. D: comparison of the sore throat between the acetaminophen groups versus the Dexamethasone groups during 24 hours after the end of the surgery

**Figure 2.**
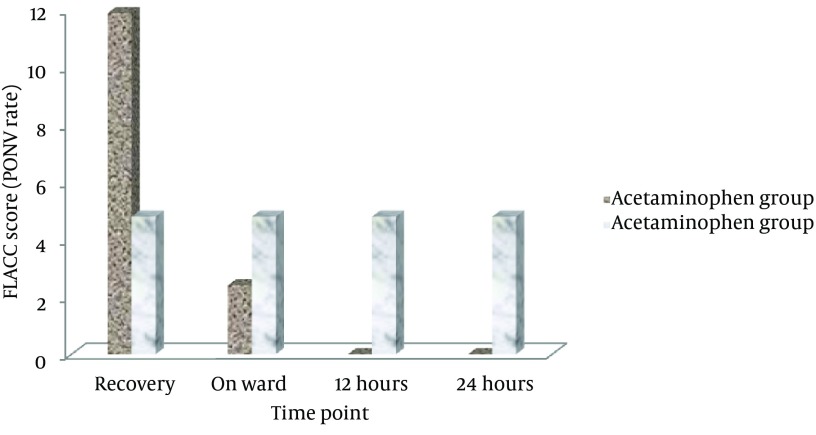
The frequency of PONV in postoperative events in the groups received intravenous acetaminophen or dexamethasone

**Figure 3.**
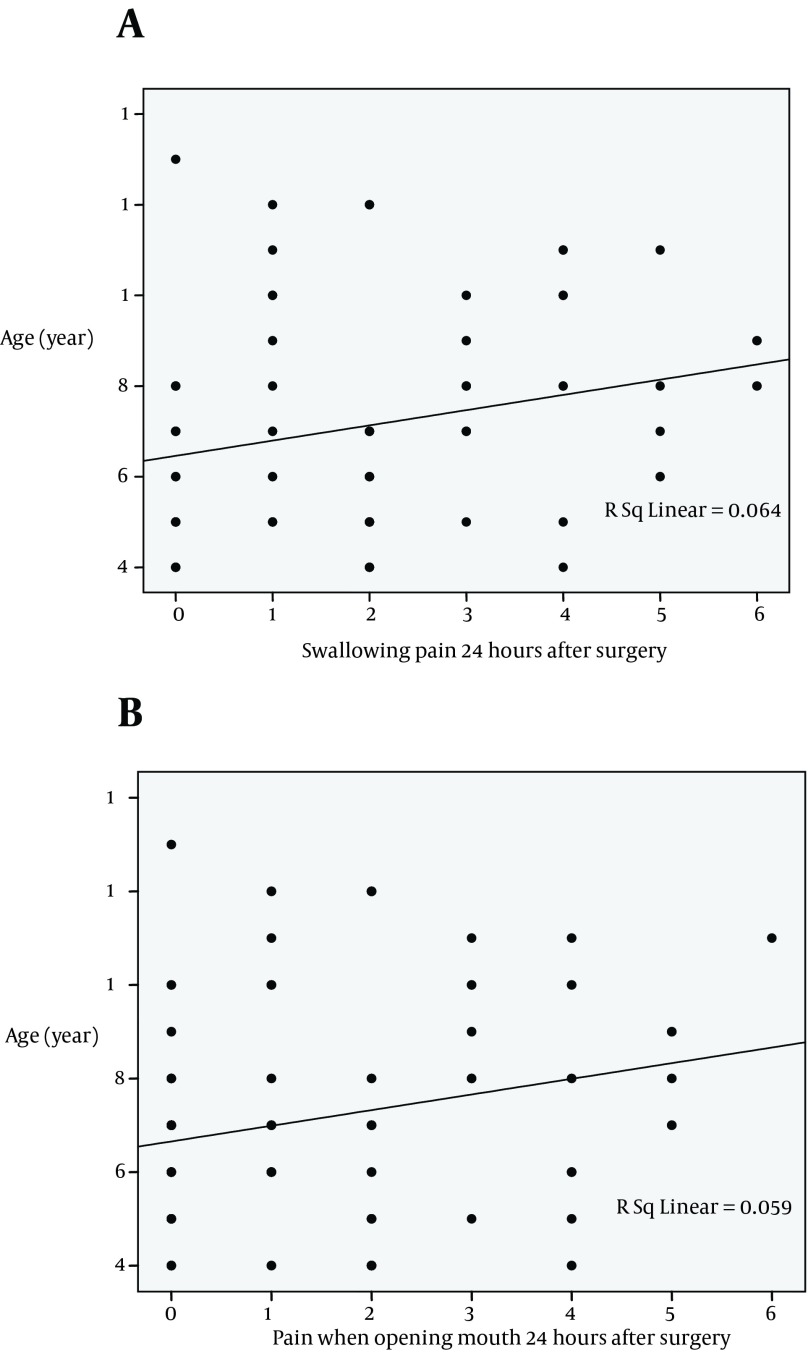
Correlation between advanced age and postoperative swallow and mouth opening pain in children undergoing tonsillectomy (A-B)

Considering trend of the changes in the severity of pointed postoperative events showed that the severity of swallowing pain, pain when opening mouth, ear pain, as well as postoperative sore throat as gradually reduced within 24 hours of tonsillectomy in both groups, but no between-group differences were observed in the trend of the severity of these events (Figure 1 A-D). Regarding prevalence of PONV, this complication was revealed in 5 patients (11.9%) in the acetaminophen group and 2 cases (4.8%) in the dexamethasone group on postoperative recovery period. This complication was appeared in 1 case in the former group and in 2 cases in another group on admission to ward. At 12 and 24 hours after surgery, PONV was only observed in 2 patients received dexamethasone, but not in those who administered acetaminophen (Figure 2). Regarding association between advanced age and postoperative pain, the severity of 24-hour swallowing pain and pain due to opening mouth was positively associated with increase of age (Figure 3 A-B); however no relationship was found between the patients’ age and severity of other postoperative complications at 24 hours after tonsillectomy.

In a total of seven patients in the dexamethasone groups and five cases of the acetaminophen groups, Amp mepridine 0.3 mg/kg PRN were used once in order to reduce pain during 24 hours after the surgery however, it didn’t cause any significant difference. (P value = 0.533). And a case of the acetaminophen group had vomited three times during 24 hours (Amp ondansetron 0.1 mg/kg PRN was used once) in which the P value = 0.314 was not significant.

## 5. Discussion

Postoperative pain and PONV remains a significant problem for children undergoing tonsillectomy, and is the commonest reason for readmission after procedures. The present study compared two different analgesic regimens including intravenous acetaminophen and dexamethasone. Although we could demonstrate a significant deceasing trend of pain severity within 24 hours of surgery in both groups, but between-group difference was not observed regarding trend of the changes in swallowing pain and pain when opening mouth. Also, more pain removing effect of dexamethasone was observed in dexamethasone group compared with acetaminophen group at 24 hours after surgery. Moreover, those who administered dexamethasone experienced severer postoperative sore throat compared to another group within 12 hours after surgery. In fact, our study emphasizes more efficacy of dexamethasone administration than intravenous acetaminophen with respect to controlling postoperative complications in children undergoing tonsillectomy. Much discomfort after tonsillectomy is potentially due to local inflammation, edema and nerve irritation. Dexamethasone is one of the most potent glucocorticoids available that can effectively suppress a basic inflammatory response to tissue injury (15, 16). Dexamethasone has been identified as a strong anti-inflammatory glucocorticoid, which also has antinociceptive influences by inhibiting the glial activation, sympathetic sprouting, production of prostaglandins, bradykinin, leukotrienes, tumor necrosis factor-a and other mediators of inflammatory hyperalgesia and central sensitization (17-20). It can also inhibit the synthesis of the COX-2 in both peripheral tissues and the central nervous system (21). Several studies, examining usage of a single bolus of intravenous dexamethasone given after induction of anesthesia in pediatric tonsillectomy patients, have found that dexamethasone effectively reduced post-tonsillectomy pain (22, 23).

Malde (24) studied the effectiveness of a single intravenous dose of dexamethasone (0.15 mg/kg) in patients aged >3 years undergoing sharp dissection snare tonsillectomy, found that dexamethasone provided significant analgesia, reduced edema and improved the quality of oral intake. It has been demonstrated that the combination of acetaminophen and a steroidal or non-steroidal anti-inflammatory drug can offer superior analgesia compared with either drug alone (25). In this parallel, dexamethasone, when used as an adjuvant to acetaminophen help to L.

In our study, although the appearance of PONV was scarce after tonsillectomy, but its rate was more observed in the group which received dexamethasone in comparison with acetaminophen administration. It has been shown that in patients with a high risk of postoperative PONV, a single prophylactic dose of dexamethasone can effective compared with placebo, without evidence of any clinically relevant toxicity (26, 27). Although similar effect has been also shown following intravenous administration of acetaminophen (28), it is believed that the best prophylaxis of PONV currently available is achieved by combining dexamethasone with acetaminophen. However, optimal doses and best administration time point of this combination need to be identified in further studies.

### 5.1. Limitation of the Study

There has been limitations in our study, for instance IV acetaminophen was given once and the half-life is 2-4 hours and duration effect 4-6 hours; we did not control for other medication use that impacts post-op pain and PONV (opioids, anti-emetics, ibuprofen); even though there is a statistical difference in post op sore throat at the first three time points, one wonders if this is really clinically significant.
